# Editorial: The Dialogue Between Forensic Scientists, Statisticians and Lawyers About Complex Scientific Issues for Court

**DOI:** 10.3389/fgene.2020.00704

**Published:** 2020-08-07

**Authors:** Sue Pope, Alex Biedermann

**Affiliations:** ^1^Principal Forensic Services, Bromley, Kent, United Kingdom; ^2^Faculty of Law, Criminal Justice and Public Administration, University of Lausanne, Lausanne, Switzerland

**Keywords:** criminal jurisprudence, expert evidence, DNA likelihood ratios, DNA evidence, principles of forensic interpretation

Courts across jurisdictions have seen a massive “scientification” of their evidential proceedings, fueled by permanent technological advances, in particular with the advent of modern DNA profiling analyses since the mid-1980s. Never before, in the history of forensic science, could analyses be extended to such small quantities of trace material, and never before have forensic experts had more powerful computational and data analytic devices at their disposal for handling the vast array of data that their analyses produce. At the same time, conceptual questions on how to assess the probative value of scientific findings have largely been settled: there is now a broad agreement that evaluating scientific evidence should adhere to the precepts of logic, balance, transparency, and robustness (e.g., Jackson, 2000; Association of Forensic Science Providers, 2009). But as much as there have been advances, modern scientific evidence has been, and is still, accompanied by challenges and contestation. What was once called the “DNA-wars” (Thompson, 1993) in the early 1990s, has developed during the last decade into refined discourses about selected aspects of scientific evidence, such as algorithmic transparency. While some of these debates are confined almost exclusively to scientific circles, they are also brought to the open by meticulous legal discussants, who care about the foundations of evidence and its ability to help discriminate between prosecution and defense views (e.g., Imwinkelried, 2017). What is more, paradoxically, much of the specialized discussion around these topics is confined to scientific journals whose deterring paywalls prevent vital information from being distributed among those practitioners—especially defense lawyers—for whom access to such information would be most beneficial. The purpose of this *Frontiers* Research Topic thus is twofold. On the one hand, the aim is to bring together a broad range of authors from various forensic science and legal disciplines (both academic and practice oriented) to elaborate on key topics that sit at the intersection between (forensic) science and the law. On the other hand, the purpose is to serve the scientific and legal community by providing this collection of contributions freely and fully accessible (open access, OA), a goal that is achieved through the *Frontiers* OA publishing model^1^.

This collection of papers focuses on so-called evaluative uses of evidence, in particular DNA evidence. That is, situations in which a potential source (i.e., reference material of known origin) for a given trace is available and the value of the results of the comparison between the trace and the reference needs to be assessed with respect to competing propositions regarding the source of the evidential material, or propositions regarding alleged activities (ENFSI, 2015; Gill et al., 2018). This is to be distinguished from so-called investigative uses of evidence, which are situations in which *no* potential source for recovered trace material is available. See, for example, Butler and Willis (2020) for a recent review on this topic, in particular investigative DNA genealogy as used, for example, in the “Golden State Killer” case. Developments in the latter field heavily rely upon large datasets generated by the expanding direct-to-customer genomic industry (e.g., Phillips, 2018).

Several papers in this collection address selected issues that affect the sound use of DNA profiling analyses in evaluative settings. Taylor et al. discuss matters that arise in connection with the use of modern computer software for biostatistical and the value of evidence computations, especially concerns raised by legal commentators. In turn, Roberts addresses general aspects of expert testimony, followed by a discussion of these aspects in the context of the use of low-template DNA profiling results by English and Northern Irish courts. Biedermann et al. and Biedermann and Hicks focus on recurrent misconceptions in the assessment of DNA profiling results, in particular the distinction between issues of source and alleged activities, and the importance of drawing this distinction carefully by acknowledging the circumstances of the case and the specific accounts provided by the prosecution and defense. The importance of these topics has recently been reiterated by guidelines published by the DNA Commission of the International Society for Forensic Genetics (Gill et al., 2018, 2020). Scientific evidence other than DNA is discussed in the legal commentaries by Caruso and Symes and Kotsoglou.

Aitken and Aitken et al. focus on statistical methodologies and concepts, in particular the likelihood ratio, which is now widely recognized as providing the most suitable framework for assessing the value of scientific evidence in a way that is logical, balanced, transparent, and robust. Both these articles address and rebut critiques (e.g., Lund and Iyer, 2017) that have recently been leveled against the likelihood ratio.

Finally, Taroni et al. discuss a case example that they consider demonstrates the gap that still exists between what academics consider sound evaluative procedures and what scientists in the field actually practice and convey to recipients of expert information. Burnier offers additional considerations regarding the same case.

## Author Contributions

Both authors have made equal contributions to the work and approved it for publication.

## Conflict of Interest

SP is a Director at the company Principal Forensic Services. The remaining author declares that the research was conducted in the absence of any commercial or financial relationships that could be construed as a potential conflict of interest.

## Footnotes

^1^This is the second *Frontiers* research topic on forensic science after “DNA, statistics and the law: a cross-disciplinary approach to forensic inference” (https://www.frontiersin.org/research-topics/1325).
